# Percutaneous lordoplasty for the treatment of severe osteoporotic vertebral compression fractures with kyphosis

**DOI:** 10.3389/fneur.2023.1132919

**Published:** 2023-07-28

**Authors:** Tengfei Song, Fan Sun, Shu Liu, Tianwen Ye

**Affiliations:** ^1^Department of Orthopedics, Changzheng Hospital, Naval Medical University, Shanghai, China; ^2^Department of Orthopedics, The Fourth Affiliated Hospital of Nangjing Medical University, Nanjing, Jiangsu, China

**Keywords:** percutaneous lordoplasty, kyphotic deformity, thoracolumbar fracture, vertebral compression fracture, osteoporosis

## Abstract

**Objective:**

The study aimed to explore the safety and effectiveness of percutaneous lordoplasty (PLP) in the treatment of severe osteoporotic vertebral compression fracture (OVCF).

**Methods:**

Included in this prospective study were patients with single-segment acute severe OVCF who were treated with PLP in our institution from July 2016 to October 2019. Patients' back pain and quality of life were assessed using the visual analog scale (VAS) and SF-36 scores. Lateral X-ray radiography of the spine was performed to measure the vertebral height, vertebral kyphotic angle, and segmental kyphotic angle, and to evaluate the outcome of fracture reduction and kyphotic correction. Intra-and postoperative complications were recorded.

**Results:**

Of the 51 included patients, 47 patients were followed up for 12 months. The VAS score decreased from preoperative 7.33 ± 1.92 to postoperative 1.76 ± 0.85 at the 12th month (*p* < 0.05), and the SF-36 score increased from preoperative 79.50 ± 9.22 to postoperative 136.94 ± 6.39 at the 12th month (*p* < 0.05). During the 1-year follow-up period, the anterior height of the vertebral body increased significantly from preoperative 10.49 ± 1.93 mm to 19.33 ± 1.86 mm (*p* < 0.05); the posterior height of the vertebral body increased insignificantly from preoperative 22.23 ± 2.36 mm to 23.05 ± 1.86 mm (*p* > 0.05); the vertebral kyphotic angle decreased significantly from preoperative 18.33° ± 11.49° to 8.73° ± 1.21° (*p* < 0.05); and the segmental kyphotic angle decreased significantly from preoperative 24.48° ± 4.64° to 11.70° ± 1.34° (*p* < 0.05). During the 1-year follow-up period, there was no significant difference in the radiologic parameters, VAS scores, and SF-36 scores, between the 1st day and the 12th month of post-operation (*P* > 0.05). No nerve damage occurred in any of the cases. Intraoperative cement leakage occurred in six cases, and the fracture of the adjacent vertebral body occurred in one case.

**Conclusion:**

PLP can well reduce the risk of fracture and achieve good kyphotic correction and may prove to be a safe, cost-effective and minimally invasive alternative option for the treatment of severe OVCF with kyphotic deformity.

## Introduction

Osteoporotic vertebral compression fracture (OVCF) is the most common complication of osteoporosis in the elderly. Approximately 20% of women older than 50 years suffer at least one segment of OVCF (1). Severe OVCF (compression >40%) often leads to kyphosis, persistent low back pain, and consequently pulmonary dysfunction, gastrointestinal dysfunction and increased mortality (2).

Surgical treatment should be considered for patients with severe OVCF with kyphotic deformity because conservative treatment is usually ineffective to relieve pain in these patients. However, the surgical management of severe OVCF with kyphosis remains a clinical challenge. Although open reduction and internal fixation can gain better fracture reduction and kyphotic correction, there are more blood loss, implant failure, and surgical site infection than that in percutaneous vertebroplasty (PVP) or percutaneous kyphoplasty (PKP) (3, 4). PVP and PKP are the most common minimally invasive surgery for OVCF (5). However, either PVP or PKP can only address a limited reduction of the fractured vertebra body and correction of the kyphotic deformity (6). A sufficient reduction of the fractured vertebral body and correction of kyphotic deformity can reduce the risk of adjacent vertebral fracture (7). The correction of kyphotic deformity also helps to reduce the occurrence of chronic low back pain. To better restore the vertebral body height and correct kyphosis, Hein et al. (8) proposed a percutaneous lordoplasty (PLP) technique for the treatment of OVCF in 2004. Some subsequent studies (9–11) have demonstrated that PLP can reduce the risk of fracture and correct the kyphotic angle of OVCF satisfactorily. However, the role of PLP in the treatment of severe OVCF with kyphosis remains undefined.

## Materials and methods

### Patients

**Figure 1:**
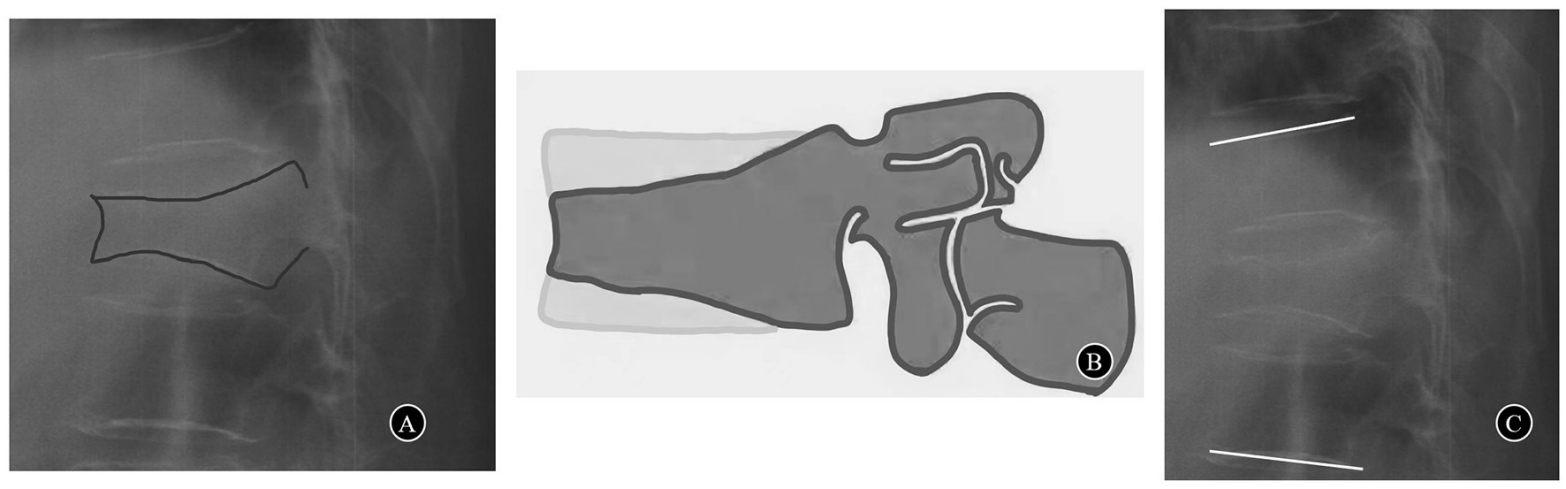
**(A)** A case of acute grade 3 OVCF (compression>40%). **(B)** Genant's classification grade 3 and severe deformity > 40% reduction of any vertebral height. **(C)** Segmental kyphotic angle.

This prospective non-randomized study included 51 consecutive patients with severe OVCF who received PLP in our institution from July 2016 to October 2019. The inclusion criteria were patients with acute grade 3 OVCF (compression >40%) according to Genant's classification (12) (Figures 1A, B) who failed to respond to conservative treatment for at least 2 weeks. The exclusion criteria were patients with fractures with neurologic deficits, posterior ligamentous complex injury and pathological fracture. The T scores in the bone mineral densities were <-2.5 in all patients. The local ethics committee authorized this study. All patients signed an informed consent form before being included in the study.

The 51 patients included 47 women and 4 men who belonged to the age group 62–79 years with a mean of 71.2 years. The locations and numbers of the fractured vertebral bodies are as follows: T10 (*n* = 3), T11 (*n* = 9), T12 (*n* = 13), L1 (*n* = 21), and L2 (*n* = 5). All patients received regular anti-osteoporosis treatments postoperatively.

### Surgical procedures

All surgical procedures were performed by the same surgeon (Dr. Ye) having a good experience in spine surgery. The principle of the PLP technique is based on indirect fracture reduction in combination with internal fixation and ligamentotaxis (9). The patient was placed in a prone position in hyperextension on a radiolucent operation table with the abdomen hung freely with a pad supporting the sternum and the pelvis. PLP was performed under general anesthesia or local anesthesia. The whole procedure was performed using a C-arm fluoroscope.

**Figure 2:**
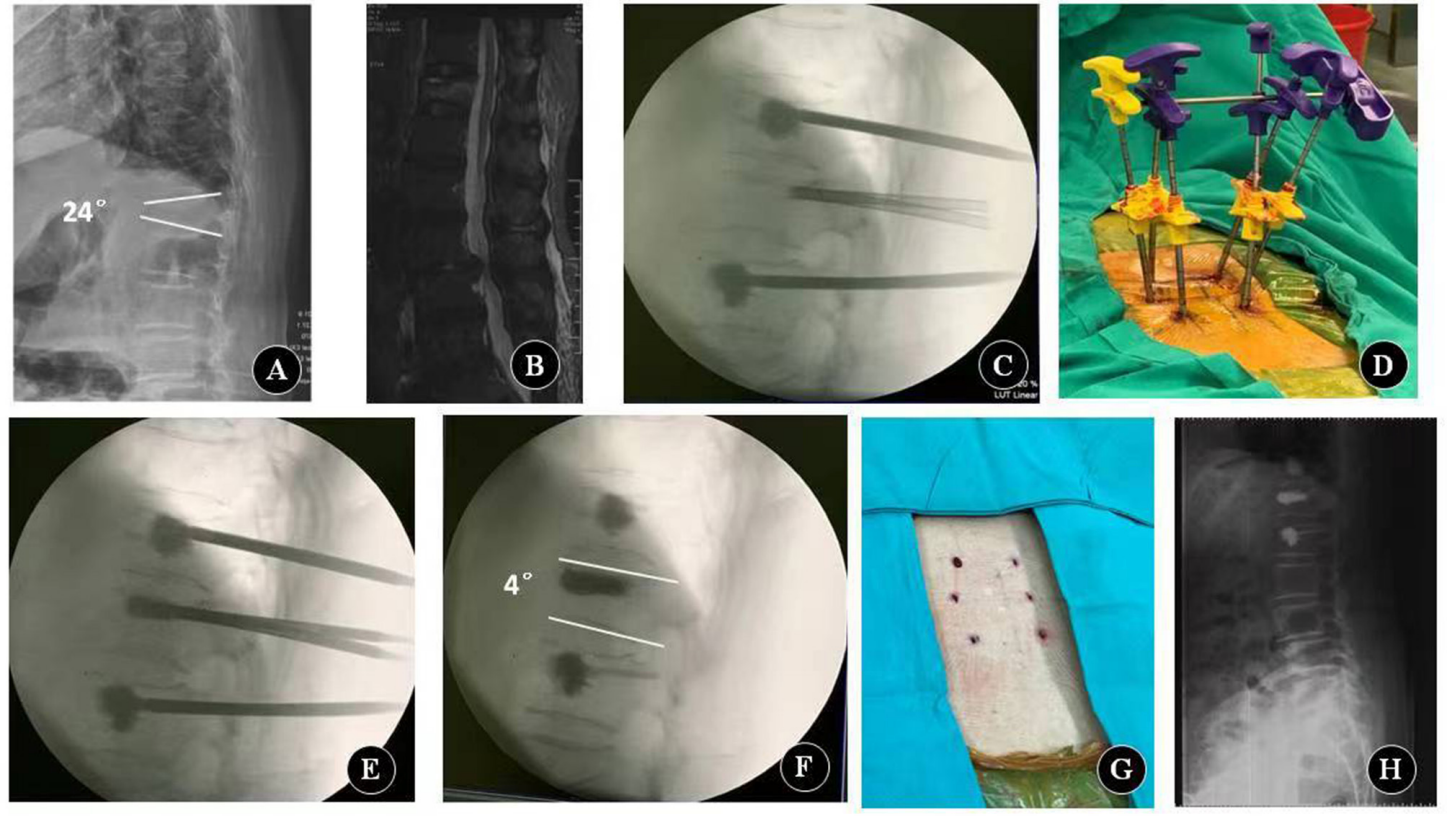
Key procedures of percutaneous lordcoplasty. **(A)** Preoperative lateral X-ray. **(B)** Preoperative sagittal MRI. **(C)** Reinforce adjacent vertebral bodies with cement. **(D)** Relevant reduction of the fractured vertebra is performed by a lordosing force. The reduction force of the cannulas is held with a cross blunt trocar. **(E)** Fractured vertebra body is augmented with cement. **(F–H)** Fluoroscopy image and appearance after the cannulas are removed.

PLP was performed as follows (Figure 2). After the stab incision, six guide K-wires of 2 mm in diameter were placed into the bilateral pedicles of the fractured and adjacent vertebrae (above and below the fractured vertebrae). The tip of the K-wires should be in front of the posterior wall of the vertebral body. The filling cannulas were placed over the wires. The tip of the cannulas should be over the ventral half of the vertebral body. After removing the guide wires, vertebroplasty of the adjacent vertebrae above and below the fractured vertebrae was performed using 1–2 ml cement at an adapted viscosity (13). When the cement had cured, a lordosing force was applied via the cannulas in the adjacent vertebrae. The fracture of the vertebral body was reduced by ligamentotaxis using the adjacent cemented vertebrae as levers and the facet joints as fulcrums. The reduction force of the cannulas was held with a cross blunt trocar. The fractured vertebra body was maintained in a reduced state using a cross trocar and then augmented by another injection of activated cement. The lordosing force was not loosened until the cement hardened. When the cement had hardened, the cannulas were released and removed.

### Clinical and radiographic evaluation

The visual analog scale (VAS) was used to quantify the patient's back pain intensity. The 36-item short-form (SF-36) was used to evaluate the quality of life of patients. Preoperative spinal X-ray radiography in the anterior–posterior and lateral view, MRI, and CT scan were performed to determine the level and severity of the fracture. Operation time, intraoperative blood loss, and the volume of bone cement were recorded. Each patient underwent standardized examinations on the day before surgery and the 1st day and the 12th month after surgery. The radiograms were obtained and analyzed to quantify the height of the vertebral body, vertebral kyphotic angle, and segmental kyphotic angle. The height of the vertebral body at the fractured and adjacent levels was measured in both the anterior and posterior portions. The vertebral kyphotic angle was ascertained by measuring the angle between the superior and inferior endplates of the fractured vertebral body on the lateral X-ray images. The segmental kyphotic angle was measured at the cephalad and caudal endplates of the vertebra as well as above and below the fractural level (Figure 1C). The extravasation of cement and adjacent vertebral fracture were also assessed on the plain X-ray films.

### Statistical analysis

Preoperative and postoperative VAS and SF-36 scores and radiological changes were compared using the Wilcoxon signed-rank test. All data are presented as the mean ± standard deviation (SD). A computerized statistical analysis was performed using SPSS software (version 20.0; SPSS Inc., Chicago, IL). The level of significance was set at a *p*-value of < 0.05.

## Results

Four patients were lost to follow-up, and the remaining forty-seven patients were followed up for a year. The mean surgical time was 87 ± 26 min. The material price of PLP was about $1,700 for every patient. Cement leakage outside the vertebral body was found in six patients. No cement leakage into the spinal canal or pulmonary cement embolism was observed in any patient. There was no intraoperative nerve damage. Anti-osteoporosis medications were applied for at least 1 year in all patients. During the 1-year follow-up period, an adjacent vertebral fracture was observed in one patient, which was managed with non-surgical measures.

The VAS and SF-36 scores were obtained before the operation and on the 1st day and 12th months of post-operation for all patients (Table 1). The VAS score of back pain decreased from preoperative 7.33 ± 1.92 to 2.11 ± 1.33 on the 1st day of post-operation and 1.76 ± 0.85 on the 12th month of post-operation. The SF-36 score increased from preoperative 79.50 ± 9.22 to 141.50 ± 8.01 on the 1st day of post-operation and 136.94 ± 6.39 on the 12th month of post-operation. Compared with the preoperative values, both VAS and SF-36 scores improved significantly on the 1st day of post-operation and the 12th month of post-operation (both *P* < 0.05), but there was no significant difference in VAS and SF-36 scores between the 1st day and the 12th month of post-operation (*P* > 0.05).

**Table 1:** VAS and SF-36 scores before and after percutaneous lordoplasty.

	**Pre-operation**	**1st day post-operation**	**12th month post-operation**
VAS	7.33 ± 1.92	2.11 ± 1.33^#^	1.76 ± 0.85^*^^※^
SF-36	79.50 ± 9.22	141.50 ± 8.01^#^	136.94 ± 6.39^*^^※^

^#^P < 0. 05, ^*^P < 0.05; ^※^P > 0.05.

# Post-operation 1^st^ day comparison with pre-operation.

* post-operation 12^th^ month comparison with pre-operation.

※ Post-operation 12^th^ month comparison with 1^st^ day.

The preoperative anterior vertebral height, posterior vertebral height, vertebral kyphotic angle, and segmental kyphotic angle were 10.49 ± 1.93 mm, 22.23 ± 2.36 mm, 18.33° ± 11.49°, and 24.48° ± 4.64°; 19.78 ± 2.65 mm, 23.26 ± 2.25 mm, 8.11° ± 1.77°, and 11.62° ± 2.17° on the 1st day of post-operation; and 19.33 ± 1.86 mm, 23.05 ± 1.86 mm, 8.73° ± 1.21°, and 11.70° ± 1.34° at the 12th-month post-operation, respectively (Table 2). The anterior height of OVCF level increased both on the 1st day and 12th month of post-operation as compared with the preoperative values (*P* < 0.05). However, the posterior height of the OVCF level did not increase significantly as compared with the preoperative value (*P* > 0.05). On the 1st day and 12th month of post-operation, the vertebral kyphotic angle and segmental kyphotic angle were decreased as compared with those before the operation (*P* < 0.05). During the 1-year follow-up period, no significant difference in the radiologic parameters was observed between the 1st day and 12th month of post-operation (*P* > 0.05).

**Table 2:** Lateral view of X-ray radiography before and after percutaneous lordoplasty.

	**Pre-operation**	**1^st^ day post-operation**	**12^th^ month post-operation**
Anterior height (mm)	10.49 ± 1.93	19.78 ± 2.65^#^	19.33 ± 1.86^*^^※^
Posterior height (mm)	22.23 ± 2.36	23.26 ± 2.25	23.05 ± 1.86^*^^※^
Vertebral kyphotic angle (°)	18.33 ± 11.49	8.11 ± 1.77^#^	8.73 ± 1.21^*^^※^
Segmental kyphotic ang^#^le (°)	24.48 ± 4.64	11.62 ± 2.17^#^	11.70 ± 1.34^*^^※^

^#^P < 0. 05, ^*^P < 0. 05, ^※^P > 0. 05.

# Post-operation 1^st^ day comparison with pre-operation.

* Post-operation 12^th^ month comparison with pre-operation.

※ Post-operation 12^th^ month comparison with 1^st^ day.

## Discussion

OVCF is the most common type of osteoporotic fracture. OVCF is classified into three types using Genant's semiquantitative method (12). Grade 3 is a severe form of OVCF causing more than 40% reduction in the anterior, mid, or posterior aspect relative to the same or adjacent vertebra on the lateral X-ray view. Severe OVCF is usually accompanied by kyphotic deformity. Addressing the kyphotic deformity, restoring the vertebral height, and correcting the kyphosis will help realign the spine. Kyphotic correction reduces the risk of adjacent vertebral fracture and chronic low back pain (7). Through spinal hyperextension in the prone position, PVP can address a limited reduction of the fractured vertebra body and correction of the kyphotic deformity. However, the poor correction of kyphosis or limited restoration of the vertebral body may lead to overloaded stress on the anterior column of the vertebral body and increase the risk of adjacent vertebral fracture after PVP (14). Theoretically, PKP can correct the kyphotic angle and restore the vertebral body height with the aid of the balloon (15), but the restoration of the kyphotic angle is limited to 6–9° due to a loss of reduction after deflating the kyphoplastic balloon and before injecting the cement during the PKP procedure (16).

In 2004, Hein et al. (8) first reported PLP for the treatment of OVCF with kyphosis. In their report, 30 OVCFs were treated by PLP, and the kyphotic angle was corrected on average by 14° or 68%. Therefore, PLP is suggested as an alternative option for restoring lordosis in OVCF cases. There are also some studies reporting that PLP could correct the vertebral kyphotic angle from 13° to 15.2° and segmental kyphotic angle from 10.0° to 11° (7, 8). In 2010, Kim et al. (11) conducted a retrospective comparative study of the clinical and radiographic outcomes of PLP and PKP for the treatment of OVCF. Their results showed that the anterior height of the vertebral body increased by 24.2% and 17.5%, the wedge angle of the vertebral body was corrected by 8.0°and 5.8°, and the kyphotic angle was corrected by 11.4° and 7.0°, respectively 3 months after the operation. Their study showed that PLP could effectively restore the anatomy of the fractured vertebral body and lower the rate of re-collapse after the procedures. In a 2-year retrospective study, PLP appeared effective in relieving pain and restoring kyphotic deformity in OVCF (10). The mean correction of the vertebral kyphotic angle was 13°, and the segmental kyphotic angle was 11° during at least a 24-month follow-up period. Jeon et al. (17) presented three cases of OVCF with kyphotic deformity treated by PLP. The wedge and kyphotic angles of the fractured vertebrae were significantly restored. Our prospective study demonstrated a remarkable vertebral reduction and kyphotic correction of PLP comparable to the previous studies.

New compression fractures in the adjacent vertebral bodies were found frequently after PVP and PKP, and the incidence ranged from 6.2 to 51.9% after PVP (14, 15, 18) and from 9.9 to 25% after PKP (19–21). Sagittal imbalance and degree of vertebral height restoration are two main risk factors of new adjacent vertebral fracture after percutaneous vertebral augmentation (14, 18). The restoration of the normal spinal sagittal alignment in the elderly with OVCF and kyphotic deformity has potential benefits. Restoring the initial vertebral body height will decrease the risk of adjacent vertebral fractures (7). Recently, Li et al. (22) had reported that aggravation of regional BMD differences induces a higher risk of adjacent vertebral fracture (AVF) after PVP surgery due to the deterioration of the local biomechanical environment. However, PLP technology reduces regional BMD differences by strengthening adjacent vertebral bodies, which may decrease AVF risks. We only found one case of new adjacent segment vertebral fracture for the good restoration of spinal sagittal alignment and vertebral height after PLP.

Cement leakage is the major concern during PLP. In PLP, multi-level vertebral bodies need to be reinforced, including the fractured vertebra and the adjacent vertebra. Theoretically, the increased number of vertebrae augmented by cement will increase the risk of cement leakage (23). However, no higher incidence of cement leakage was observed in both our and other studies (9–11). Kim et al. (11) reported no significant difference in cement leakage between PLP and PKP. Hoppe et al. (10) reported a 10% rate of cement leakage into the segmental vein or paraspinous tissue but no leakage into the spinal canal or pulmonary cement embolism in 69 cases of PLP. Orler et al. (9) observed individual cases of minor leakage during the LP procedure without leading to any significant postoperative clinical signs. In this study, the rate of cement leakage was 7.8%, and no leakage into the spinal canal or pulmonary cement embolism was observed, indicating that PLP is a safe augmentation technique according to the present studies.

The cost associated with each technique is an important factor to be considered before surgery. The excessive cost of PKP depends on the price of the implant balloon. PLP is more cost-effective compared with PKP (9–11, 17, 24). In Sweden, the material price of PKP is six times that of PLP (3,600 *vs*. 600 Euros) (24). In South Korea, the cost of treatment for PKP is $3,644 *vs*. $1,525 for PLP (11). The price of the material for PLP is approximately one-third of that of PKP (9). In our hospital, the material price of PKP and PLP is about $3,800 and $1,700, respectively. PLP is a lower-cost minimally invasive surgery and is more easily accepted by low-income patients with OVCF. Therefore, PLP may be applied excessively in developing countries in future.

Both the clinical and radiographic outcomes of PLP in the treatment of severe OVCF are satisfactory. Nevertheless, there are several limitations in the present study. First, the number of cases treated with the LP technique is relatively small, which may decrease the generalizability of the results presented here. Second, the follow-up period is not long enough. we only conducted a 1-year follow-up data analysis of the radiographic measures, which may bring a statistical bias in the evaluation of the correction of kyphotic deformity after the PLP procedure. Third, no analysis of osteoporosis drugs was conducted in this study. The types of drugs are not clear for each patient, which may cause limitations in the analyses. In fact, we have noted that no significant difference in the radiologic parameters was observed between the 1st day and the 12th month of post-operation in the 1-year follow-up. However, the radiologic parameters tend to get worse after 1 year. Insufficient anti-osteoporosis treatment in some patients may be one of the reasons. Finally, this study is a prospective case study without a control group, which is considered a level IV of evidence. Therefore, it is prudent to make a conclusion that PLP possesses more advantages such as vertebral height restoration and kyphotic correction than PVP and PKP. Larger cohort prospective randomized controlled studies with longer follow-up periods are required to verify the results and conclusion of this technique reported in our study.

In conclusion, PLP is a safe, cost-effective, and minimally invasive alternative option to relieve pain, restore vertebral body height, and correct kyphotic deformity in patients with severe OVCF.

## Data availability statement

The original contributions presented in the study are included in the article/supplementary material, further inquiries can be directed to the corresponding author.

## Ethics statement

The studies involving human participants were reviewed and approved by Medical Ethics Committee, Shanghai Changzheng Hospital. The patients/participants provided their written informed consent to participate in this study.

## Author contributions

All authors listed have made a substantial, direct, and intellectual contribution to the work and approved it for publication.

## References

[1] Kendler DL Bauer DC Davison KS Dian L Hanley DA Harris ST. Vertebral fractures: clinical importance and management. Am J Med. (2016) 129:221.e1–10. 10.1016/j.amjmed.2015.09.020 26524708

[2] Popa I. The variability of vertebral body volume and pain associated with osteoporotic vertebral fractures: conservative treatment versus percutaneous transpedicular vertebroplasty. Int Orthop. (2017) 41:963–8. 10.1007/s00264-017-3409-2 28161853

[3] Chen JW Chanbour H Roth SG Stephens BF Abtahi AM Zuckerman SL. How much blood loss is appropriate for a 2- to 3-level posterior lumbar fusion. Int J Spine Surg. (2023) 17:241–9. 10.14444/8423 36828635 PMC10165640

[4] Guo H-Z Tang Y-C Guo D-Q Ma Y-H Yuan K Li Y-X. Pedicle screw fixation in single-level, double-level, or multilevel posterior lumbar fusion for osteoporotic spine: a retrospective study with a minimum 2-year follow-up. World Neurosurg. (2020) 140:e121–8. 10.1016/j.wneu.2020.04.198 32376379

[5] Schupfner R Stoevelaar HJ Blattert T Fagan D Fransen P Marcia S. Treatment of osteoporotic vertebral compression fractures: applicability of appropriateness criteria in clinical practice. Pain Physician. (2016) 19:E113–20. 10.36076/ppj/2016.19.E113 26752479

[6] El-Fiki M. Vertebroplasty, kyphoplasty, lordoplasty, expandable devices, and current treatment of painful osteoporotic vertebral fractures. World Neurosurg. (2016) 91:628–32. 10.1016/j.wneu.2016.04.016 27072339

[7] Ottardi C La Barbera L Pietrogrande L Villa T. Vertebroplasty and kyphoplasty for the treatment of thoracic fractures in osteoporotic patients: a finite element comparative analysis. J Appl Biomater Funct Mater. (2016) 14:e197–204. 10.5301/jabfm.5000287 27032865

[8] Heini PF Orler R. Kyphoplasty for treatment of osteoporotic vertebral fractures. Eur Spine J. (2004) 13:184–92. 10.1007/s00586-003-0654-4 14986073 PMC3468137

[9] Orler R Frauchiger LH Lange U Heini PF. Lordoplasty: report on early results with a new technique for the treatment of vertebral compression fractures to restore the lordosis. Eur Spine J. (2006) 15:1769–75. 10.1007/s00586-006-0108-x 16724212

[10] Hoppe S Budmiger M Bissig P Aghayev E Benneker LM. Lordoplasty: midterm outcome of an alternative augmentation technique for vertebral fractures. J Neurosurg Spine. (2016) 24:922–7. 10.3171/2015.10.SPINE151016 26895528

[11] Kim SB Jeon TS Lee WS Roh JY Kim JY Park WK. Comparison of kyphoplasty and lordoplasty in the treatment of osteoporotic vertebral compression fracture. Asian Spine J. (2010) 4:102–8. 10.4184/asj.2010.4.2.102 21165313 PMC2996621

[12] Genant HK Wu CY van Kuijk C Nevitt MC. Vertebral fracture assessment using a semiquantitative technique. J Bone Miner Res. (1993) 8:1137–48. 10.1002/jbmr.5650080915 8237484

[13] Belkoff SM Mathis JM Jasper LE Deramond H. The biomechanics of vertebroplasty. The effect of cement volume on mechanical behavior. Spine. (2001) 26:1537–41. 10.1097/00007632-200107150-00007 11462082

[14] Ma X Xing D Ma J Wang J Chen Y Xu W. Risk factors for new vertebral compression fractures after percutaneous vertebroplasty: qualitative evidence synthesized from a systematic review. Spine. (2013) 38:E713–22. 10.1097/BRS.0b013e31828cf15b 23429687

[15] Savage JW Schroeder GD Anderson PA. Vertebroplasty and kyphoplasty for the treatment of osteoporotic vertebral compression fractures. J Am Acad Orthop Surg. (2014) 22:653–64. 10.5435/JAAOS-22-10-653 25281260

[16] Eck JC Nachtigall D Humphreys SC Hodges SD. Comparison of vertebroplasty and balloon kyphoplasty for treatment of vertebral compression fractures: a meta-analysis of the literature. Spine J. (2008) 8:488–97. 10.1016/j.spinee.2007.04.004 17588820

[17] Jeon TS Kim SB Park WK. Lordoplasty: an alternative technique for the treatment of osteoporotic compression fracture. Clin Orthop Surg. (2011) 3:161–6. 10.4055/cios.2011.3.2.161 21629479 PMC3095789

[18] Park JS Park YS. Survival analysis and risk factors of new vertebral fracture after vertebroplasty for osteoporotic vertebral compression fracture. Spine J. (2021) 21:1355–61. 10.1016/j.spinee.2021.04.022 33971326

[19] Fribourg D Tang C Sra P Delamarter R Bae H. Incidence of subsequent vertebral fracture after kyphoplasty. Spine. (2004) 29:2270–6; discussion 2277. 10.1097/01.brs.0000142469.41565.2a 15480139

[20] Spross C Aghayev E Kocher R Röder C Forster T Kuelling FA. Incidence and risk factors for early adjacent vertebral fractures after balloon kyphoplasty for osteoporotic fractures: analysis of the SWISSspine registry. Eur Spine J. (2014) 23:1332–8. 10.1007/s00586-013-3052-6 24197481

[21] Ko BS Cho KJ Park JW. Early adjacent vertebral fractures after balloon kyphoplasty for osteoporotic vertebral compression fractures. Asian Spine J. (2019) 13:210–5. 10.31616/asj.2018.0224 30481974 PMC6454291

[22] Li J Xie Y Sun S Xue C Xu W Xu C. Regional differences in bone mineral density biomechanically induce a higher risk of adjacent vertebral fracture after percutaneous vertebroplasty: a case-comparative study. Int J Surg. (2023) 109:352–63. 10.1097/JS9.0000000000000273 36912508 PMC10389488

[23] Cheng Y Cheng X Wu H. Risk factors of new vertebral compression fracture after percutaneous vertebroplasty or percutaneous kyphoplasty. Front Endocrinol. (2022) 13:964578. 10.3389/fendo.2022.964578 36120447 PMC9470857

[24] Luginbühl M. Percutaneous vertebroplasty, kyphoplasty and lordoplasty: implications for the anesthesiologist. Curr Opin Anaesthesiol. (2008) 21:504–13. 10.1097/ACO.0b013e328303be62 18660662

